# Causes and Types of Acidosis*Read before the Surgeons Club of the Atlanta Medical College, Emory University.

**Published:** 1916-03

**Authors:** Edgar G. Ballenger, Omar F. Felder

**Affiliations:** Atlanta, Ga.; Atlanta, Ga.


### CAUSES AND TYPES OF ACIDOSIS*

By EDGAR G. BALLENGER, M. D., F. A. C. S. and
OMAR F. FELDER, M. D., Atlanta, Ga.

Acidosis may occur as a result of a sufficient increase in the acid intake or an increase in the bodily production of acid in the process of metabolism with the excretion of it being normal or below normal· On the other hand, acidosis may result from a normal production or intake of acid when its elimination and neutralization persists below normal. Fortunately the remedy is efficacious whether or not we are able to find the exact cause of the acidosis. We know of many things which cause an increase

*Read before the Surgeons Club of the Atlanta Medical College, Emory University.

in the acid, such as anesthetics, alcoholism, insufficient oxygen, the so-called nerve or mental strain, profound anxiety, infections, excess of acid food and acid liquids, excess of protein food, certain gastro-intestinal disturbances, diabetes, etc. Naturally from such diverse causes we have very wide variations in the degree and character of the acidosis. The health of animal as well as plant life demands an alkaline medium. The activities of life are constantly producing acid as a by-product of energy production. Exertion, emotion, and anything which produces an energy transformation causes an increase in acid as a by-product. An increase in adrenin also results. By experiments it has been shown that the injection of carbon dioxide causes an increase in respiration and an increase in the output of adrenin without other symptoms. The length of time a patient is able to hold his breath has been advanced as an indicator of the degree of acidosis. In well marked acidosis a patient can hold his breath only fifteen to thirty seconds. Crile says that "in a greater or less degree acidosis is present in every abnormal condition of the body in which can be traced excessive kinetic activations, and maintainance of the normal potential alkalinity of the body is of vast clinical importance." The brain, lungs, adrenals, and liver are concerned in the neutralization and elimination of acids, the brain governing the mechanism by which the actual processes are accomplished. For a number of years we have been much interested in the subject of acidosis because of its importance in dealing with elderly men who require careful preparation to tide them over the increase in acids incident to the operations they may require, such as prostactectomy, stone in the bladder, or kidney, etc., where the renal operative risk is unusually great. That there is a distinct type of nephritis caused by a chronic acidosis has been clearly shown by Martin H. Fischer and confirmed by many others. That it is a very common cause of nephritis we are convinced. That a healthy or diseased kidney will better withstand the extra strain placed upon it by anesthetics and the shock incident to the operation we are equally certain. Therefore since we cannot remove certain causes inseparably connected with operations we should combat the acidosis by the preliminary alkalies, lemon juice, glucose and copious draughts of water. We cannot explain to you the reasons why anesthetics produce acidosis. The essential thing to remember is that it does increase the acids and that the excess of acid produces nausea, vomiting, restlessness and causes damage to the kidneys and liver.

Alcoholism, as previously stated, causes an acidosis; this becomes apparent the day following an excessive intake and produces a fairly constant symptom complex of acidosis. The exact method of its production we are unable to explain. Salvarsan also causes acidosis with nausea, vomiting, nervousness and renal irritation. Alkalies, plenty of water and previous purgation prevent or lessen these harmful effects. Just how it produces the acidosis again is not clear. That the acidosis is the cause of the nausea and vomiting seems proved by the relief usually promptly afforded by equal parts of honey, citrate of soda and brandy, in one drachm doses every three ro four hours as needed.

That insufficient oxygen can cause an acidosis was many years ago shown by Fischer who strapped guinea pig's chest and saw the urine become more acid and the production of albumin and casts in the urine. The same results follow ligation of the renal artery. The urine became normal more quickly when alkalies were administered than when they were not given. This would seem to indicate that an excess of acid was produced and that the local acidosis was the cause of the albumin and casts.

Infections of various kinds cause acidosis. This, of course, is not the only toxin to be combatted but being one that is easily controlled it should always receive our attention. One of the common causes of backache, especially when associated with infections, fever, anesthetics, and concentrated, highly acid urine is a colloidal swelling of the renal substance caused by the acidosis which may be proved by giving the patient sufficient citrate of soda or lemon juice to render the urine alkaline. Acidosis during pregnancy is an important subject though it is entirely outside of our work and we are not familiar with it. We think though alkalies are indicated when albumin and casts first appear and perhaps serious damage to the liver and kidneys resulting ultimately in eclampsia might be obviated. There is an important type of acidosis caused by or at least associated with gastrointestinal toxemia and infections in children.

The brevity of this paper has made it necessary to omit many interesting facts concerning this important subject which will be more fully discussed in the other papers tonight.

805-9 Healey Building.

